# Long-term efficacy and tolerability of quetiapine in patients with schizophrenia who switched from other antipsychotics because of inadequate therapeutic response—a prospective open-label study

**DOI:** 10.1186/s12991-014-0039-6

**Published:** 2015-01-22

**Authors:** Naoki Hashimoto, Atsuhito Toyomaki, Minoru Honda, Satoru Miyano, Nobuyuki Nitta, Hiroyuki Sawayama, Yasufumi Sugawara, Keiichi Uemura, Noriko Tsukamoto, Tsukasa Koyama, Ichiro Kusumi

**Affiliations:** Department of Psychiatry, Hokkaido University Graduate School of Medicine, 060-8638, North 15, West 7, Kita-ku, Sapporo, Japan; Child and Adolescent Psychiatry, Department of Psychiatry University of California, 401 Parnassus Ave, Box 0984-F, San Francisco, CA 94143 USA; Honda Memorial Hospital, 061-1364, 619-1, Shimoshimamatsu Eniwa, Hokkaido, Japan; Teine Hospital, 006-0816, 8-15, Maeda 6-jo 13-chome, Teine-ku, Sapporo, Japan; Sapporo Hanazono Hospital, 064-0915, 1-30, South 15, West 15, Chuo-ku, Sapporo, Japan; San-ai Hospital, 059-0463, 24-12, Nakanoboribetsu-cho Noboribetsu, Hokkaido, Japan; Psychiatric Medical Center, Sapporo City General Hospital, 060-0011 1-1, North 11, West 13, Chuo-ku, Sapporo, Japan; Department of Psychiatry, Hakodate Watanabe Hospital, 042-0932, 1-31-1, Yunokawa Hakodate, Hokkaido, Japan; Oyachi Hospital, 004-0041, 7-10, Oyachi-higashi 5-chome, Atsubetsu, Sapporo, Japan

**Keywords:** Schizophrenia, Quetiapine, Switching, Antipsychotic, Negative symptom, Cognitive impairment, Treatment resistance

## Abstract

**Background:**

While the frequency and importance of antipsychotic switching in patients with schizophrenia, there is insufficient evidence with regard to switching strategy. Quetiapine is one of the drugs of choice for switch because of its unique receptor profile. However, there were no data on the long-term clinical and neurocognitive effect of quetiapine in patients who had responded inadequately to prior antipsychotics. The purpose of this study is to examine the long-term efficacy and tolerability of quetiapine in patients with schizophrenia who switched from other antipsychotics because of inadequate therapeutic response. We hypothesized that quetiapine would show long-term effectiveness in broad symptom dimensions including negative and neurocognitive symptoms while having good tolerability.

**Methods:**

Twenty-nine subjects with schizophrenia who did not respond to their current monotherapy of antipsychotic or who could not tolerate the treatment were switched to quetiapine and assessed at baseline and at 3, 6, and 12 months. The outcome measures included the brief assessment of cognition in schizophrenia (BACS), the Positive and Negative Syndrome Scale (PANSS), the Clinical Global Impressions Scale (CGI), the Schizophrenia Quality of Life Scale Japanese version (JSQLS), the Athens Insomnia Scale (AIS), and the Drug Attitude Inventory with 30 items (DAI-30). The Drug-Induced Extrapyramidal Symptoms Scale (DIEPSS), HbA1c, prolactin (PRL), and body weight were also evaluated.

**Results:**

Statistically significant improvements were observed in all subscores of the PANSS, the GAF, and the symptoms and side effects subscale of the JSQLS, the DIEPSS, the AIS, and the PRL level, and nearly significant improvements were observed in the DAI-30. Quetiapine monotherapy was associated with significant improvement in the verbal memory test, even after controlling for the practice effect. Although quetiapine was well tolerated, three subjects dropped out because of the worsening of the psychotic symptoms and two additional subjects dropped out because of somnolence.

**Conclusion:**

In this open-label, single-arm study of 29 patients, quetiapine improved both the clinical symptoms and the neurocognitive impairment in chronic schizophrenia patients who failed to respond to prior antipsychotic treatment.

**Electronic supplementary material:**

The online version of this article (doi:10.1186/s12991-014-0039-6) contains supplementary material, which is available to authorized users.

## Background

The antipsychotic medications have been a fundamental part of the treatment of schizophrenia. Although the benefit of antipsychotic treatment is evident especially about the psychotic symptoms, inadequate therapeutic response is common in this disorder [1,2]. If a patient does not respond well to the administered antipsychotic, and if the possibility of misdiagnosis or non-compliance is excluded, a switch of the currently prescribed antipsychotic is an often employed step [3]. In fact, it is estimated that switching because of suboptimal antipsychotic efficacy or tolerability occurs in 30%–50% of patients a year in outpatient clinic [4].

Although little is known about the optimal clinical strategies for switching, it seems to be rational to choose a new compound with a different receptor binding profile [3]. From this point of view, quetiapine is one of the strong candidates for some patients because of its unique receptor profile. Quetiapine demonstrates a relatively high affinity for the 5HT_2A_ receptor and relatively low affinity for the D_2_ receptor [5]. Such a low affinity for the D_2_ receptor is thought to be appropriate if the first compound was characterized by high affinities to dopamine receptors such as risperidone or first-generation antipsychotic (FGA) [3]. Furthermore, the high 5HT_2A_/D_2_ ratio leads to an overall increase in dopaminergic activity in the prefrontal cortex, and enhanced prefrontal activity is shown to be associated with the alleviation of negative symptoms and the improvement of cognitive function [6]. It is also important because the cognitive and negative symptoms are often refractory to antipsychotic treatment [7,8] and become the cause of inadequate response. Collectively, this evidence suggests that a study examining the long-term efficacy of quetiapine on negative as well as cognitive symptoms after switching from other antipsychotics might be quite valuable.

There were several studies that examined the long-term efficacy and tolerability of the quetiapine comparing with placebo [9] or risperidone [10-13]. Although the difference in the modes of dosage should be noted (oral risperidone [10,11] and risperidone long-acting injection [12,13], oral quetiapine immediate [11] and extended release [9,12]), in general, quetiapine showed long-term effectiveness similar to [11] or less than risperidone [10], a positive relapse prevention effect better than placebo [9] and equal to risperidone [12] except for its ability to achieve remission, which was less than risperidone [12,13]. When focused on studies that switched over to quetiapine, Larmo and his colleagues (2005) showed significant improvement in the Positive and Negative Syndrome Scale (PANSS) positive, negative, and general psychopathology subscales after switching to quetiapine in patients who were previously treated with low-dose haloperidol, risperidone, or olanzapine [14]. Recently, Chue et al. (2013) reported another open-label, prospective study to evaluate long-term clinical benefits of switching to quetiapine extended release from an oral antipsychotic in patients with schizophrenia. In this study, majority of the subjects switching from other antipsychotics to quetiapine due to insufficient efficacy or insufficient tolerability showed clinical benefits [15]. In all of these studies, quetiapine showed good long-term tolerability with little extrapyramidal side effects and hyperprolactinemia [10,14], and most common adverse events were somnolence and dizziness [11-15].

With regard to pharmacological treatment of the cognitive impairment, recent large sample naturalistic studies showed that the magnitude of cognitive improvement does not differ between first- and second-generation antipsychotics or between any two second-generation antipsychotics, if the dose of first-generation antipsychotics were appropriate [16-18]. However, in a randomized, double-blind 52-week comparison on neurocognitive function in early psychosis, quetiapine showed greater improvement than both olanzapine and risperidone on measures of verbal fluency and digit symbol coding test and than olanzapine on continuous performance test at Week 12 [19]. The superiority of quetiapine compared to other second-generation antipsychotics were also shown in a recent meta-analysis of two double-blind and one open-label studies [20]. However, there has been no data on the long-term neurocognitive effects of quetiapine in patients who had responded inadequately to prior antipsychotics.

In this study, we examined the long-term efficacy and tolerability of quetiapine in patients with schizophrenia who switched from other antipsychotics because of inadequate therapeutic response. We hypothesized that quetiapine would show long-term improvement in broad symptom dimensions including negative and neurocognitive symptoms while having good tolerability.

## Method

### Participants

This is a prospective, open-label study. The eligible subjects were schizophrenia patients aged 20 years or more, who need switch of antipsychotic because of an inadequate response (cognitive impairment, negative, positive, or general psychopathology symptoms) or intolerance for their current antipsychotic medication. The diagnosis according to the DSM-IV-TR and judgment about an inadequate response and/or poor tolerability were made by treating psychiatrists, who had at least 6 years of clinical experience. These eligible criteria are in consistent with the SPECTRUM trial [14]. Patients who had a history of seizure disorder, dementia, diabetes mellitus, history of substance misuse including alcohol abuse, or other significant laboratory results were excluded from the study. The subjects who were currently suicidal and women who were pregnant or breastfeeding were also excluded.

The study was conducted between January 2008 and December 2012 at nine clinical sites in Hokkaido, Japan (one university hospital, three general hospital, and five psychiatric hospitals). This study was approved by the ethics committees at Hokkaido University Hospital and has been carried out in accordance with the Declaration of Helsinki. We provided detailed explanations regarding the study procedures and the potential risk and benefits of pharmacotherapy to the eligible subjects. All participants voluntarily provided their written informed consent.

### Procedure

The trial began with a cross-titration period of 1 month to allow a gradual transition, followed by an 11-month follow-up phase during which quetiapine could be flexibly dosed. During the cross-titration period, a dose of quetiapine was titrated at a target dose of 400 mg/day, while the previous antipsychotic was gradually discontinued. During the follow-up period, the maximum dose of quetiapine was 750 mg/day. The concomitant use of psychotropics, such as anxiolytics, mood stabilizers, and antidepressants, was permitted, and the daily dosages of all psychotropics were recorded throughout the study. Hypnotics were tapered off if possible after the assessment at 6 months. The concomitant use of drugs for parkinsonism, which included anticholinergic agents, was permitted for the first 3 months, and it was also tapered off after the assessment at 3 months; continuous use was permitted if necessary. Non-psychoactive medications for stable conditions that were already taken by the subject before entry into the study were allowed to continue.

### Clinical assessments

The assessments were performed at four points (baseline and 3, 6, and 12 months after entry). The baseline assessment had been done before cross-titration. Clinical improvement was examined using the Global Assessment of Functioning (GAF) and the PANSS [21]. Quality of life was assessed by the Schizophrenia Quality of Life Scale Japanese version (JSQLS) [22]. Vital signs and laboratory data, including prolactin and hemoglobin A1c (HbA1c), were checked at each assessment point. At every visit, adverse events were carefully checked via spontaneous reports from the patient and observation by the same treating psychiatrist. In addition, assessment of the extrapyramidal symptoms using the Drug-Induced Extrapyramidal Signs Scale (DIEPSS) [23] and sleep difficulty assessment using the Athens Insomnia Scale (AIS) [24] were done at each assessment.

### Assessment of neurocognition

For the neurocognitive assessment, we adopted the Brief Assessment of Cognition in Schizophrenia (BACS) [25,26], which is a brief set of tests designed to derive a composite score for neurocognitive functioning. The score from each test of the BACS was standardized by creating z score whereby the mean score of the healthy subjects were set to zero and the standard deviation was set to one. The composite score was then calculated by averaging all of the z scores of the six primary measures. The mean and standard deviation of normal Japanese individuals were derived from the published data of 709 normal Japanese subjects [27]. To compensate for practical effects, we recruited normal subjects and implemented the BACS four times at the same time interval as our schizophrenia subjects (baseline and 3, 6, and 12 months after). For the control group, ten subjects (five females) with no history of a DSM-IV Axis I disorder were recruited from the community. None of the control subjects had a neurological disorder or a first-degree relative with a DSM-IV Axis I disorder, nor were any of them receiving psychotropic medications. The mean age of the participants was 27.3 (SD = 2.3) years, and the duration of education was 18.2 (1.5) years. Both of which were significantly different from those of our schizophrenia subjects (age 50.7 (14.3), Wilcoxon test, *P* < 0.01; duration of education 12.9 (1.7), Wilcoxon test, *P* < 0.01) (Additional file 1).

### Statistical analyses

We compared the baseline categorical variables using *χ*^2^ or Fisher exact tests and examined the differences in the continuous variables among the follow-up time points using with repeated measure analysis of variance (rANOVA). Bonferroni correction was applied for multiple comparisons between each pair of subgroups. The last available assessment was carried forward if the actual data were not available (LOCF). Safety evaluations were based on all included patients treated with the studied medication except for three subjects who were omitted from all analyses because of incomplete data. Data were obtained from 28 subjects for PANSS and DIEPSS; 26 subjects for weight, HbA1c, GAF, and AIS; 25 subjects for BACS; 24 subjects for prolactin and DAI-30; and 24 subjects for JSQLS.

For the neurocognitive study, the baseline measures of the demographic data and the BACS z scores were compared between the schizophrenic subjects and the control group specifically recruited for the practice effect using the Wilcoxon test for continuous variables and the Fisher’s exact test for categorical variables. In the next step, analysis of covariance (ANCOVA) was used to detect the group (schizophrenia and normal subjects) x time (baseline to 3, 6, and 12 months) interaction, as well as the interaction as a factor of the baseline pretest score or age.

All comparison tests were two-tailed. Differences among groups were considered statistically significant if the *P* value was less than 0.05.

## Results

### Patient description and baseline demographics, severity of illness, and previous treatments

A total of 32 patients signed the informed consent forms, but three of them were omitted from the analysis including safety analysis because of incomplete data and the remaining 29 patients were included. Seventeen subjects entered the study due to treatment resistance, six entered due to treatment intolerance, and six were recruited due to both treatment resistance and treatment intolerance. The subjects were medicated as follows: risperidone (n.16); haloperidol (n.8); olanzapine (n.3); aripiprazole (n.1); blonanserin (n.1). The mean chlorpromazine equivalent dosage for the previous treatment was 438.0 (s.d. 242.3) mg/day.

Twenty-seven subjects completed the 3-month evaluation, 26 finished the 6-month assessment, and 22 reached the 12-month mark. Three patients dropped out because of worsening of psychotic symptoms, two dropped out because of somnolence, and one dropped out because of alopecia. Another one subject was discontinued due to a change of address. The last observation was carried forward for the analysis of patients who were dropped from the study. The baseline demographic data, the severity of illness, and previous treatment are shown in Table 1. There were no significant differences in the demographic and clinical profiles between the subjects who completed the study and those who did not.

**Table 1:** **Baseline demographic and clinical profiles**

	**Completed (** ***n*** **= 22)**	**Withdrawn (** ***n*** **= 7)**	***P*** ^**a**^
Sex (male/female)	10/12	3/4	0.59
Age, year	52.1 (14.4)	46.5 (14.1)	0.49
Clinical subtype			0.49
Paranoid	18	7	
Disorganized	1	0	
Catatonic	1	0	
Residual	2	0	
Reason for switching			0.60
Treatment resistant	13	3	
Treatment intolerant	5	3	
Resistant and intolerant	4	1	
Education, year			
Subject	12.9 (1.8)	13.0 (1.0)	0.59
Father	12.3 (2.6)	10.5 (1.9)	0.28
Mother	12.0 (2.6)	10.0 (1.7)	0.25
Previous antipsychotics			0.13
Haloperidol	5	3	
Risperidone	12	4	
Others	5	0	
Average dose of previous antipsychotics (mg/day)	445.9 (230.6)	409.2 (303.8)	0.63

Values are expressed as the mean (SD) unless otherwise indicated.

^a^Differences in two groups with *P* values calculated by the Wilcoxon test or the *χ* ^2^ test (sex, clinical subtype, reason for switching, and previous antipsychotics).

### Efficacy

The patients who switched to quetiapine had significant improvements in all subscores of the PANSS, the JSQLS score for symptoms and side effects, and GAF (Table 2). AIS score also improved significantly. The reason to switch (resistant vs intolerant) did not affect the change of PANSS positive (repeated ANOVA, effect of times x reason to switch, F(3,19) = 1.14, *P* = 0.36) and negative scores (repeated ANOVA, effect of times x reason to switch, F(3,19) = 1.00, *P* = 0.41).

**Table 2:** **Changes in clinical measurements**

	**Baseline**	**3 months**	**6 months**	**12 months**	**Repeated ANOVA** ^**a**^	
					***F***	***P***
Average dose of QTP (mg/day)	-	379.6 (231.7)	418.5 (243.4)	438.6 (261.7)	-	-
Weight (kg)	56.9 (12.2)	57.6 (12.4)	57.0 (11.6)	57.3 (10.7)	F(3,75) = 0.44	0.64
HbA1C (%)	4.97 (0.32)	4.95 (0.37)	4.98 (0.35)	4.89 (0.32)	F(3,75) = 1.32	0.28
PANSS						
Positive symptoms	18.1 (4.7)	15.7 (4.8)	16.4 (54.2)	15.3 (4.1)	F(3,81) = 5.35	<0.01
Negative symptoms	23.5 (5.2)	21.5 (5.5)	20.8 (6.2)	19.6 (6.5)	F(3,81) = 11.40	<0.01
General pathological symptoms	39.8 (10.2)	36.8 (9.3)	36.8 (10.5)	34.7 (10.1)	F(3,81) = 7.83	<0.01
GAF	42.4 (14.7)	50.3 (12.7)	51.7 (14.0)	51.6 (15.6)	F(3,75) = 10.54	<0.01
AIS	5.0 (3.1)	4.9 (3.6)	3.5 (2.9)	3.7 (3.0)	F(3,75) = 3.35	0.04
DIEPSS	6.4 (4.9)	3.9 (3.2)	3.1 (2.8)	3.0 (2.9)	F(3,81) = 13.74	<0.01
Prolactin (ng/ml)	33.1 (26.2)	17.8 (20.8)	21.0 (27.0)	19.9 (28.0)	F(3,69) = 4.04	0.02
DAI-30	−8.6 (3.2)	−5.0 (7.1)	−6.8 (5.2)	−6.3 (5.9)	F(3,69) = 2.78	0.07
JSQLS						
Psychosocial	37.9 (13.5)	37.2 (11.7)	35.0 (11.6)	35.3 (12.2)	F(3,66) = 1.42	0.25
Motivation	21.6 (2.9)	20.2 (4.0)	19.9 (2.8)	19.7 (3.6)	F(3,66) = 2.23	0.11
Symptoms and side effects	16.7 (4.5)	14.7 (4.3)	14.4 (4.6)	14.9 (5.4)	F(3,66) = 3.20	<0.05

Values are expressed as the mean (SD) unless otherwise indicated.

^a^ *Post hoc* analyses with Bonferroni correction: (pairs of comparison not specified here were all with *P* values of >0.05, *BL* baseline, *3 m* 3 months, *6 m* 6 months, *12 m* 12 months) PANSS positive symptoms: BL vs 12 m (*P* = 0.02); PANSS negative symptoms: BL vs 3 m (*P* = 0.01), BL vs 6 m(*P* = 0.03), BL vs 12 m (*P* < 0.01), 3 m vs 12 m (*P* = 0.02); PANSS general pathological symptoms: BL vs 3 m (*P* = 0.02), BL vs 12 m (*P* < 0.01), 6 m vs 12 m (*P* = 0.03); GAF: BL vs 3 m (*P* < 0.01), BL vs 6 m (*P* < 0.01), BL vs 12 m (*P* < 0.01); Athens Insomnia Scale: BL vs 6 m (*P* = 0.03); DIEPSS: BL vs 3 m (*P* < 0.01), BL vs 6 m (*P* < 0.01), BL vs 12 m (*P* < 0.01); prolactin: BL vs 3 m (*P* < 0.01), BL vs 12 m (*P* < 0.01); JSQLS symptoms and side effects: BL vs 3 m (*P* = 0.03).

*HbA1c* hemoglobin A1c, *PANSS* Positive and Negative Symptoms Scale, *GAF* Global Assessment of Functioning, *AIS* Athens Insomnia Scale, *DIEPSS* Drug-Induced Extrapyramidal Signs Scale, *DAI-30* Drug Attitude Inventory with 30 dichotomous items, *JSQLS* Schizophrenia Quality of Life Scale Japanese version.

### Safety and tolerability

Quetiapine significantly improved the DIEPSS score and the prolactin level throughout the study period. (Table 2) The weights and HbA1c did not increase during the treatment with quetiapine. The amelioration of the adverse events was also shown in the improvement of the symptoms and side effects subscale of the JSQLS. Three patients dropped out because of worsening of psychotic symptoms. Two additional patients were dropped out from the study because of somnolence. The DAI-30 score showed a trend for increase but did not reached statistical significance (*P* = 0.07).

### Changes in the neurocognitive battery test

**Figure 1:**
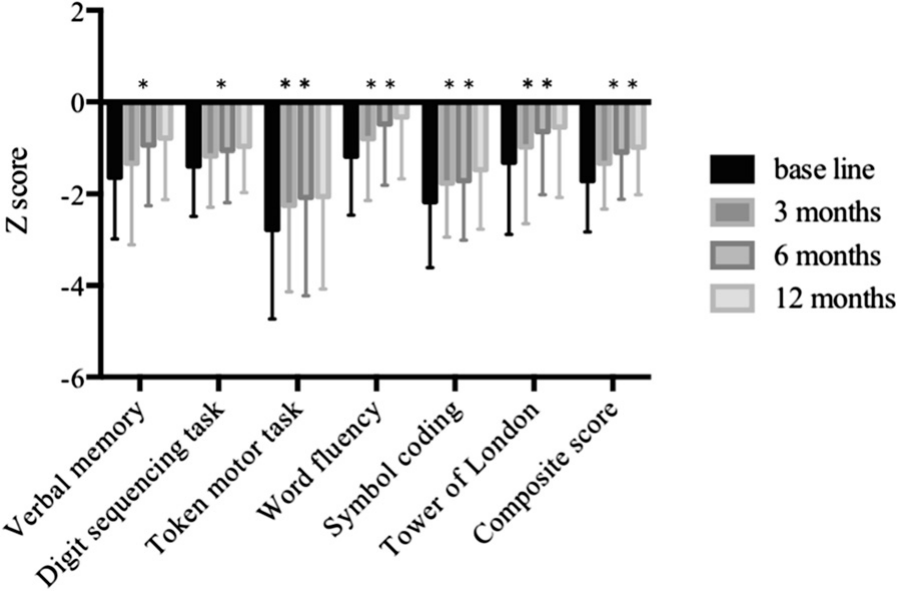
**The change of the scores of Brief Assessment of Cognition in Schizophrenia (BACS).** Results of each test were standardized by setting the mean of the normal dataset to zero and the SD to one. *BL* baseline, *3 m* 3 months, *6 m* 6 months, *12 m* 12 months. *P < 0.05, **P < 0.01, repeat ANOVA. *Post hoc* analyses were conducted with Bonferroni correction. Verbal memory: BL vs 6 m, *P* = 0.04, BL vs 12 m, *P* < 0.01; digit sequencing task: BL vs 12 m, *P* = 0.01; word fluency: BL vs 6 m, *P* = 0.03, BL vs 12 m, *P* < 0.01; symbol coding: BL vs 3 m, *P* = 0.02, BL vs 12 m, *P* < 0.01; Tower of London: BL vs 6 m, *P* = 0.01, BL vs 12 m, *P* < 0.01; composite score: BL vs 3 m, *P* = 0.43, BL vs 6 m, *P* < 0.01, BL vs 12 m, *P* < 0.01, 3 m vs 12 m, *P* = 0.04. *P* > 0.05 for all other paired comparisons.

Additional file 1 summarizes the demographic characteristics and BACS scores at the baseline of the patients and controls. There were significant differences in the age, the duration of education, and all the scores on the BACS. The z scores of the BACS in our schizophrenic subjects ranged from −2.78 (token motor test) to −1.18 (word fluency), which were similar to previous studies. In the next step, ANCOVA was implemented to detect the group (schizophrenia and normal subjects) x time (baseline to 3, 6, and 12 months) interaction, as well as the interaction as a factor of the baseline pretest score or age (Figure 1, Additional file 2). In this analysis, we found a significant effect of time on verbal memory, the token motor test, and the composite score. Furthermore, we found a significant interaction of time x group in the verbal memory test. *Post hoc* analyses using ANOVA with Bonferroni correction revealed that both normal subject group [F(3,27) = 3.67, (*P* = 0.03)] and schizophrenia subject group [F(3, 72) = 4.30, (*P* < 0.01)] showed significant increase of the score. The effect size (Cohen’s *d*) of the improvement between baseline and 12 months was 0.21 for normal subjects and 0.64 for schizophrenia subjects, which showed that the improvement of verbal memory by quetiapine therapy was greater than the practice effect of normal subjects.

## Discussion

This study was a prospective, 1-year open-label study to evaluate the long-term effect of quetiapine on the clinical symptoms and cognitive performance in patients with schizophrenia who had an inadequate response or who poorly tolerated their previous antipsychotic medication. The total drop-out rate was 24.1% (7/29), which is consistent with prior open-label study [14,18]. Statistically significant improvements were observed on all subscales of the PANSS, the GAF, and the symptoms and side effects subscale of the JSQLS, the DIEPSS, the AIS, and the prolactin level, and nearly significant improvements were observed in the DAI-30. Regarding the neurocognitive performance, quetiapine therapy showed significant improvement in verbal memory test even after controlling the practice effect. Overall, as expected, quetiapine showed long-term improvement in broad symptom dimensions including negative and neurocognitive symptoms with good tolerability. To the best of our knowledge, this is the first study, albeit preliminary, showing long-term neurocognitive benefits of switching to quetiapine in patients that showed inadequate response to prior antipsychotics.

In this trial, the patients who switched to quetiapine had significant improvements of broad range of symptoms as demonstrated by the changes in the all subscores of the PANSS, the JSQLS score for symptoms and side effects, and GAF. A clinical benefit was also shown for insomnia reflected by the improvement of AIS score.

In concordance with our hypothesis, negative symptoms were ameliorated in addition to the positive and general pathological symptoms. In the study by Larmo and his colleagues, when quetiapine was switched from risperidone, the improvement of the negative and general psychopathological symptoms was more prominent than that of the positive symptom [14]. In our study, most patients had been treated by haloperidol (27.6%) or risperidone (51.7%) before registration for our study. By replacing these treatments with quetiapine, which has a much weaker affinity for the D2 receptor, we successfully achieved amelioration of the negative symptoms. This is consistent with the previous report which showed that higher postsynaptic D2 blockade could mimic certain negative symptoms [28].

In accordance with our other hypothesis, quetiapine therapy also showed significant improvement in verbal memory, even after controlling for practice effects. This improvement continued for a year. Impaired verbal memory is associated with poor community functioning and a poor response to psychosocial rehabilitation programs [29], and the improvement of verbal memory is particularly important for the cognitive remediation strategy [30]. However, we should evaluate this result with maximum caution because 1) a switch design study is not suitable for comparing the cognitive remediation effects of antipsychotics [8], 2) the improvement of cognitive disturbance may be due to the improvement of clinical symptoms and/or extrapyramidal side effects in part [31], and 3) there is a possibility of ceiling effect in the data of normal control.

About the tolerability, switching to quetiapine induced a decrease in the prolactin levels, improvement of the extrapyramidal symptoms, and a decrease in the use of anticholinergics. At the baseline and at each time point, the number of the subjects who were prescribed with anticholinergics and the mean daily biperiden equivalent dose changed as follows: baseline [n.13, dose. 3.7 (SD = 2.2) mg/day]; 3 months [n.10, dose 3.3 (2.1) mg/day]; 6 months [n.7, dose 3.7 (2.4) mg/day]; 12 months [n.7, dose 3.7 (2.4) mg/day]. Weight gain and glucose intolerance were not apparent in our patients. Somnolence and worsening of the psychotic symptoms were noticeable adverse events, and this finding is consistent with previous studies [11,13-15]. However, the sedative effect of quetiapine improved the insomnia of our subjects, and this was reflected in the improvement of the AIS score. Tolerability findings were consistent with the trending improvement in DAI, although it did not reach statistical significance level. This may be due to the small sample size.

There are several limitations in our study. First, the small sample size of our study may limit the ability to detect moderate to small changes. As a consequence of the small sample size, we could not perform subgroup analyses, such as an analysis according to the prior antipsychotics. Second, because this was an open-label study with no control group except for the neurocognitive battery test, our study was prone to selection and observer bias. There was no indication of spontaneous recovery. Third, our normal control for the neurocognitive battery test was much younger and much longer educated than our schizophrenia subjects, and the score of the neurocognitive battery test was higher in the normal control group, though we used these factors as covariants in the statistical analysis. A younger age results in a better practice effect in general, and this age difference had never shown a favorable effect in the schizophrenia patient group. However, there is a possibility of ceiling effect in the score of our normal subject and the improvement of neurocognitive battery test in this study should be examined carefully, as we discussed above. Fourth, we did not have an objective measurement of treatment adherence, although the results of the DAI-30 indirectly suggested that their adherence was good throughout the study period.

## Conclusion

In this open-label, single-arm study of 29 patients, we found that quetiapine improved both the clinical symptoms and the neurocognitive impairment in chronic schizophrenia patients who switched from other antipsychotics because of inadequate response. While the frequency and importance of antipsychotic are switching in patients with schizophrenia, there is insufficient evidence with regard to switching strategy. We hope that further studies seeking better antipsychotic switching strategies in clinical practice will be continued.

## Additional files

Additional file 1:
**Demographic characteristics and BACS z scores of schizophrenic and normal subjects.**
Additional file 2:
**Change of the BACS z scores and the results of ANCOVA controlled for age.**

## Abbreviations

AIS: Athens Insomnia Scale
ANCOVA: analysis of covariance
BACS: brief assessment of cognition in schizophrenia
CGI: Clinical Global Impressions Scale
DAI-30: Drug Attitude Inventory with 30 dichotomous items
DIEPSS: Drug-Induced Extrapyramidal Signs Scale
HbA1c: hemoglobin A1c
JSQLS: Schizophrenia Quality of Life Scale Japanese version
LOCF: last observation carried forward
PANSSs: Positive and Negative Syndrome Scales
rANOVA: repeated measure analysis of variance
